# Chimeric Zika Viruses Containing xrRNA1 Elements of Divergent Flaviviruses Retain the Ability to Replicate in Vertebrate and Mosquito Cells and Produce Subgenomic Flaviviral RNA

**DOI:** 10.3390/v18091023

**Published:** 2026-09-15

**Authors:** Chandra S. Tangudu, Wichan Dankaona, Nile L. Stoltz, Tyler R. Simpkins, Bradley J. Blitvich

**Affiliations:** 1 Department of Veterinary Microbiology and Preventive Medicine, College of Veterinary Medicine, Iowa State University, Ames, IA 50011, USA; ctangudu@pantheravax.com (C.S.T.); retro_89@hotmail.com (W.D.); nstoltz@iastate.edu (N.L.S.); trsimpk@iastate.edu (T.R.S.); 2 Department of Anatomy, Faculty of Veterinary Medicine, Kasetsart University, Bangkok 10900, Thailand

**Keywords:** flavivirus, Zika virus, subgenomic flavivirus RNAs, xrRNA, chimeric virus

## Abstract

Members of the *Flaviviridae* encode XRN1-resistant RNA structures (xrRNAs) within their 3′ untranslated regions (UTRs) that stall the cellular 5′→3′ exoribonuclease 1 (XRN1), resulting in the accumulation of subgenomic flavivirus RNAs (sfRNAs). Zika virus (ZIKV) contains two such elements, xrRNA1 and xrRNA2, which generate sfRNA1 and sfRNA2, respectively. A third sfRNA species (sfRNA3) has also been described. Building on evidence that sequences within the orthoflavivirus UTRs modulate host range, we replaced the xrRNA1 element in the ZIKV genome with xrRNA1 or xrRNA1-like elements from nine evolutionarily divergent flaviviruses and assessed the *in vitro* host ranges of the resulting chimeric viruses to determine whether xrRNA1 contributes to host specificity. We selected mosquito-borne, tick-borne, insect-specific, and vertebrate-specific orthoflaviviruses, together with representative viruses from the *Pestivirus*, *Pegivirus*, and *Hepacivirus* genera. All genetic exchanges yielded chimeric viruses that replicated in both vertebrate (Vero) and mosquito (C6/36) cells. Wild-type ZIKV replicated to substantially higher titers than all chimeric viruses in Vero cells but showed no consistent replication advantage in C6/36 cells. All chimeric viruses produced sfRNA1, whereas detection of sfRNA2 and sfRNA3 varied according to the substituted xrRNA1 sequence and host cell type, indicating that heterologous xrRNA1 substitution can alter downstream sfRNA production in a host cell-dependent manner. In summary, replacement of the ZIKV xrRNA1 element with evolutionarily divergent xrRNA1 or xrRNA1-like sequences did not alter *in vitro* host range or abolish sfRNA1 production, but substantially reduced viral fitness in vertebrate cells.

## 1. Introduction

The family *Flaviviridae* (order *Amarillovirales*) has traditionally contained four recognized genera: *Hepacivirus*, *Orthoflavivirus*, *Pegivirus*, and *Pestivirus* [1,2]. From a public health perspective, *Orthoflavivirus* is the most important genus because it contains several globally important pathogens, such as all four serotypes of dengue virus, Japanese encephalitis virus, West Nile virus (WNV), yellow fever virus, and Zika virus (ZIKV) [3,4,5,6,7]. Orthoflaviviruses can be broadly classified according to their host range and transmission cycle. Many orthoflaviviruses, including all the aforementioned pathogens, are maintained in transmission cycles involving arthropod vectors (primarily mosquitoes or ticks) and vertebrate hosts and are commonly referred to as mosquito- and tick-borne flaviviruses (MBFVs and TBFVs). In contrast, other orthoflaviviruses are restricted to either insects or vertebrates [8,9,10]. Insect-specific orthoflaviviruses (ISFs) can be divided into two groups: classical ISFs (cISFs), which form a distinct phylogenetic lineage, and dual-host affiliated ISFs (dISFs), which phylogenetically cluster with MBFVs despite their inability to replicate in vertebrate cells [9,10]. Vertebrate-specific orthoflaviviruses, commonly referred to as no known vector viruses (NKVs), are maintained through direct transmission among bats or rodents [8]. Viruses in the genera *Hepacivirus*, *Pegivirus*, and *Pestivirus* are maintained primarily through direct horizontal transmission among vertebrate hosts, with no known arthropod vector involvement [2,11,12,13,14]. Although the *Flaviviridae* Study Group of the International Committee on Taxonomy of Viruses recently proposed a major taxonomic revision that would reorganize these viruses into three families and 12 genera [2], their website continues to display the earlier four-genus classification and therefore, the traditional classification is used throughout this study.

Viruses in the family *Flaviviridae* have a broadly conserved genomic architecture. Their genomes are typically single-stranded, positive-sense RNAs of approximately 9 to 13 kb [2,12,14,15]. Flaviviral genomes usually contain a single long open reading frame (ORF) flanked by highly structured 5′ and 3′ untranslated regions (UTRs) that have essential roles in viral translation and replication. Translation of the ORF produces a polyprotein that undergoes co- and post-translational cleavage by viral and host proteases to generate the mature viral proteins. Although this overall genomic strategy is conserved across the family, the number, identity, and arrangement of the structural and nonstructural proteins differ among genera.

The cellular 5′→3′ exoribonuclease 1 (XRN1) plays a central role in eukaryotic gene regulation by degrading defective, uncapped, or otherwise aberrant RNAs [16,17]. XRN1 also contributes to antiviral defense, as it can inhibit viral replication by degrading viral RNAs, including those of flaviviruses [18]. However, a growing number of flaviviruses have been shown to contain XRN1-resistant RNA structures (xrRNAs) within their 3′ UTRs that stall exonuclease progression, thereby preventing complete degradation of the viral RNA [19,20,21]. Stalling of XRN1 results in the accumulation of subgenomic flavivirus RNAs (sfRNAs), which are known to modulate host antiviral responses and enhance viral replication and transmission [22,23,24,25,26,27,28,29,30,31].

Subgenomic flavivirus RNAs were first identified in the brains of mice infected with Murray Valley encephalitis virus (MVEV) and have subsequently been detected in mammalian and mosquito cells infected with many other orthoflaviviruses [32,33,34,35,36,37,38,39,40,41,42,43]. Although most sfRNA research has focused on orthoflaviviruses, recent studies have identified xrRNA-like structures in the genomes of hepaciviruses, pegiviruses, and pestiviruses, suggesting that XRN1 resistance and structured RNA-mediated nuclease stalling are conserved strategies across the family *Flaviviridae* [44,45]. The number and arrangement of xrRNAs vary among flaviviruses, with individual xrRNAs capable of independently stalling XRN1 and generating distinct sfRNA species that may differ in abundance and function [21,36,41,42,46].

The ZIKV genome contains two xrRNA structures, designated xrRNA1 and xrRNA2 (also referred to as SL-I and SL-II, respectively) [35]. Stalling of XRN1 at xrRNA1 generates sfRNA1, whereas stalling at xrRNA2 generates sfRNA2 [27,35,47,48,49]. A third, smaller RNA species (sfRNA3) has also been detected in some cell types. The mechanism underlying sfRNA3 biogenesis is unclear, but it has been proposed to result from XRN1 stalling at a downstream dumbbell structure [27]. The three-dimensional structure of ZIKV xrRNA1 has been resolved by X-ray crystallography, as has that of MVEV xrRNA2 [34,35]. Disruption of the ZIKV xrRNA1 structure through the introduction of specific mutations abolishes sfRNA1 production [35,47,48]. Previous studies have demonstrated that sfRNA1-deficient ZIKV replicates less efficiently than wild-type virus in the brains and placentas of type I interferon receptor knockout mice [48]. In addition, sfRNA1-deficient ZIKV replicates more efficiently than wild-type ZIKV in mosquito cells but less efficiently in human cells [47].

To identify the genetic determinants of flavivirus host specificity, we and others have generated chimeric viruses by exchanging selected genomic regions among representative viruses from the arthropod-borne, insect-specific, and vertebrate-specific orthoflavivirus groups and characterized their *in vitro* host ranges [50,51,52,53,54,55,56,57,58,59,60,61]. These studies have highlighted the importance of flaviviral UTRs in host specificity, as revealed by work showing that ZIKV loses its ability to replicate in vertebrate cells when its 5′ and 3′ UTRs are replaced with those of dISFs [51,60]. However, the specific RNA structures and sequence elements within the flaviviral UTRs that modulate host specificity remain poorly defined. We are systematically investigating how specific secondary structures within the 5′ and 3′ UTRs individually contribute to flavivirus host specificity. Previously, we investigated the viral promoter (5′ stem loop A) and 3′ stem loop (3′ SL), which occur in the 5′ and 3′ UTRs, respectively [51,52]. xrRNA1 was selected as another candidate determinant of host specificity because of its established role in sfRNA production and the documented effects of sfRNAs on virus–host interactions and viral replication in vertebrate and mosquito cells. Building upon these earlier studies, here we investigated whether xrRNA1 contributes to flavivirus host specificity by replacing the ZIKV xrRNA1 element with xrRNA1 or xrRNA1-like elements from diverse orthoflaviviruses and representative viruses of the *Hepacivirus*, *Pegivirus*, and *Pestivirus* genera.

## 2. Methods

### 2.1. Cell Lines and Virus

*Aedes albopictus* (C6/36) and *Chlorocebus sabaeus* (African green monkey) kidney (Vero) cells were obtained from the American Type Culture Collection (Manassas, VA, USA). C6/36 cells were cultured in Liebovitz’s L-15 medium (Thermo Fisher Scientific, Carlsbad, CA, USA), while Vero cells were cultured in Dulbecco’s modified Eagle medium (Thermo Fisher Scientific). All media were supplemented with 10% fetal bovine serum, 2 mM L-glutamine, 100 U/mL penicillin and 100 μg/mL streptomycin. Dulbecco’s modified Eagle medium was also supplemented with sodium bicarbonate to a final concentration of 0.075% (*w*/*v*) (0.75 g/L). For experiments requiring minimal cell growth, the serum concentration was reduced to 2%. C6/36 cells were incubated at 28 °C, while Vero cells were incubated at 37 °C with 5% CO_2_. ZIKV (strain PRVABC59) was obtained from the virus reference collection at the University of Texas Medical Branch in Galveston, Texas, USA, and had undergone no more than three additional passages before use in this study.

### 2.2. Synthetic xrRNA1 Sequences

**Figure 1:**
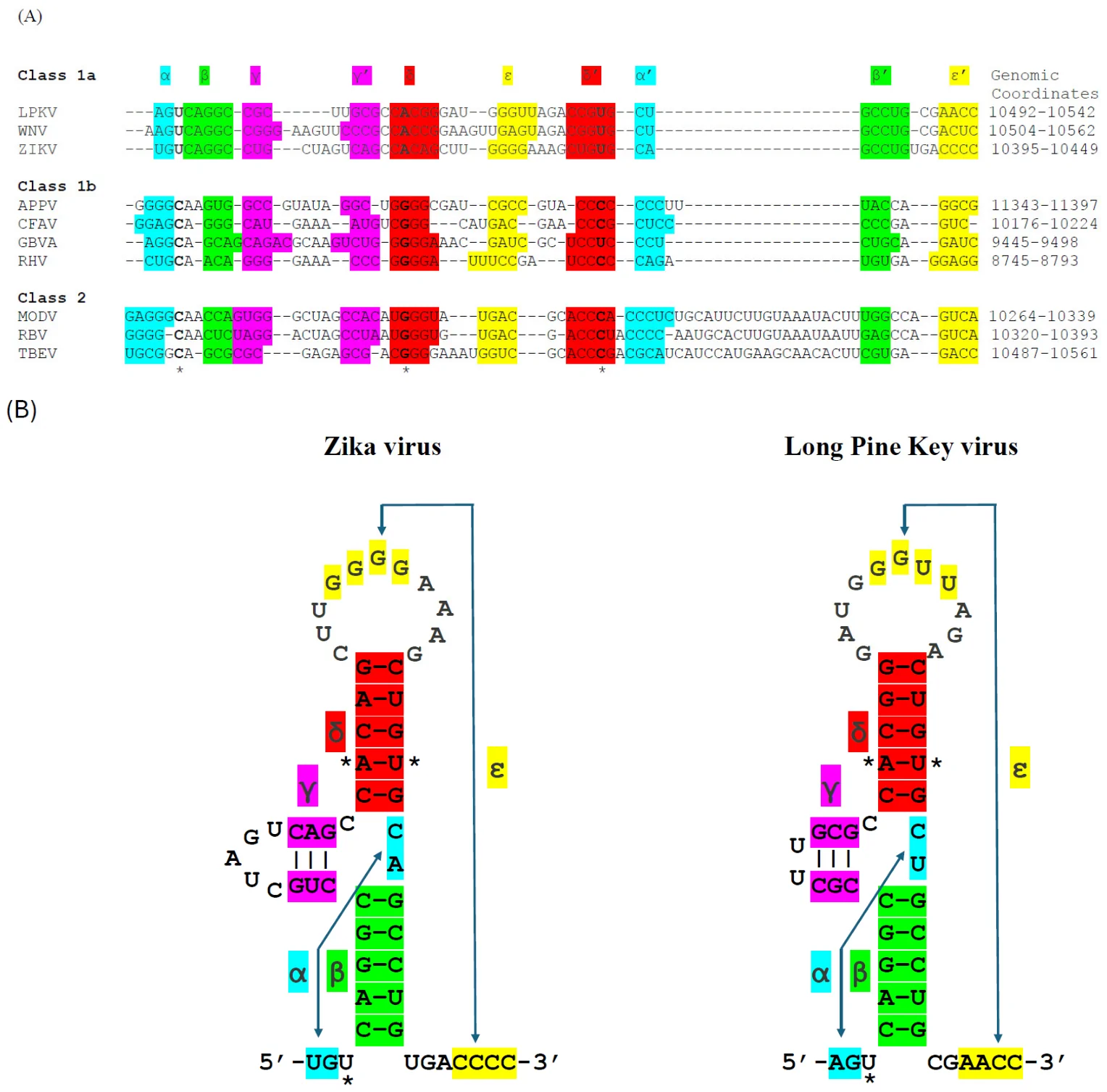
Verified or predicted xrRNA1 or xrRNA1-like elements of the viruses selected for this study. (**A**) Nucleotide sequences of the xrRNA1 or xrRNA1-like elements of the viruses selected for this study and (**B**) manually modeled secondary structures of the xrRNA1 elements of Zika virus and Long Pine Key virus. The five regions predicted to participate in stem-forming interactions (α, β, γ, δ and ε) are shaded blue, green, pink, red, and yellow, respectively. Nucleotides predicted to participate in base-triplet interactions are shown in bold in (**A**) and indicated by an asterisk in both (**A**,**B**). Genbank Accession Nos. are as follows: atypical porcine pestivirus (MH102210.1), cell fusion agent virus (NC_001564.2), GB virus-A (NC_001837.1), Long Pine Key virus (MZ090957.1), Modoc virus (NC_003635.1), Rio Bravo virus (JQ582840.1), rodent hepacivirus (KC411807.1), tick-borne encephalitis virus (MN520112.1), West Nile virus (AF196835.2), and Zika virus (KU501215.1).

Double-stranded DNA fragments encoding the xrRNA1 or xrRNA1-like elements of selected flaviviruses were synthetically produced (Integrated DNA Technologies, Coralville, IA, USA). Six representative orthoflaviviruses were selected: cell fusion agent virus (CFAV; a cISF), Long Pine Key virus (LPKV; a dISF), Modoc virus (MODV; a rodent-associated NKV), Rio Bravo virus (RBV; a bat-associated NKV), tick-borne encephalitis virus (TBEV; a TBFV), and WNV (a MBFV). One representative virus from each of the remaining genera within the family *Flaviviridae* was also included: atypical porcine pestivirus (APPV; *Pestivirus*), GB virus-A (GBVA; *Pegivirus*), and rodent hepacivirus (RHV; *Hepacivirus*). The nucleotide sequences and genomic coordinates of all xrRNA1 and xrRNA1-like elements, together with the GenBank Accession numbers of the corresponding viral genomes, are provided (Figure 1).

The nucleotide sequences and predicted secondary structures of most xrRNA1 and xrRNA1-like elements used in this study have been reported previously [34,40,44]. For CFAV, MODV, RBV, TBEV, and ZIKV, the xrRNA1 sequences described by Dilweg and colleagues [40] were used. For WNV, the xrRNA1 sequence reported by Pijlman and colleagues [32] was used, except that two nucleotide substitutions were introduced so that it matched the North American prototype strain (NY99-flamingo382-99) rather than the Kunjin strain. The xrRNA1-like elements of APPV, GBVA, and RHV were identical to those reported by Dilweg and colleagues [44]. In contrast, the LPKV xrRNA1 sequence has not been previously described and was identified by sequence alignment with previously characterized flaviviral xrRNA1 and xrRNA1-like elements (Figure 1).

Each synthetic xrRNA1 and xrRNA1-like sequence was flanked by 37 bp of ZIKV-derived sequence at both termini to facilitate the subsequent Gibson assembly reactions. The 5′ end of each fragment contained ZIKV sequence corresponding to genomic positions 10,358 to 10,394, which is located immediately upstream of the xrRNA1 element in the native ZIKV genome. The 3′ end of each fragment contained ZIKV sequence corresponding to genomic positions 10,450 to 10,485, which is located immediately downstream of the xrRNA1 element in the native ZIKV genome.

### 2.3. Plasmids

Viral chimeras were assembled using three previously described plasmids, pUC19-F1, pUC19-F2, and pUC19-F3, which together encompass the complete ZIKV genome as overlapping fragments [50]. The pUC19-F1 plasmid contains a modified *Orgyia pseudotsugata* multicapsid nucleopolyhedrosisvirus immediate-early 2 promoter (OpIE2-CA) upstream of ZIKV genomic sequence spanning nucleotides 1 to 3460. The pUC19-F2 plasmid contains ZIKV genomic sequence spanning nucleotides 3413 to 8071. The final plasmid, pUC19-F3, contains ZIKV genomic sequence spanning 8016 to 10,807, followed by a hepatitis delta virus (HDV) antigenomic ribozyme sequence and simian virus 40 (SV40) polyadenylation signal [51,52].

### 2.4. Chimera Production

Viral chimeras were constructed by replacing the xrRNA1 element in the ZIKV genome with the corresponding xrRNA1 or xrRNA1-like sequences of each selected donor virus. To generate each chimera, pUC19-F3 was first modified to remove the native xrRNA1 element. This was achieved by PCR amplification of pUC19-F3 using a forward primer (ZIKV-3F) and reverse primer (ZIKV-3R) targeting genomic positions 10,449–10,470 and 10,374–10,394, respectively. The resulting amplicon lacked the native xrRNA1 element, while retaining the remainder of the plasmid sequence. Gibson assembly reactions were then performed by incubating the amplicon with one of the synthetic xrRNA1 or xrRNA1-like fragments. Assembly was facilitated by the 37 bp ZIKV-derived overlapping sequences at both ends of each synthetic fragment. The resulting plasmids were transformed into chemically competent *E. coli* cells and a subset of colonies was screened by Sanger sequencing. Clones containing the expected viral sequences without mutations were selected. Primer sequences used for chimera construction are provided (Table 1).

Following replacement of the ZIKV xrRNA1 element in pUC19-F3 with each donor xrRNA1 or xrRNA1-like sequence, the nine resulting plasmids were used as templates in separate PCR reactions designed to amplify all the viral sequences, while excluding the pUC19 vector backbone. All of these reactions used the same primer pair, designated as Chimera-3F and -3R (Table 1). Similarly, pUC19-F1 and pUC19-F2 were used as templates in separate PCR reactions designed to amplify all the viral sequences while excluding the pUC19 vector backbone, using primer pairs Chimera-1F and -1R and Chimera-2F and -2R, respectively (Table 1).

### 2.5. Gibson Assembly, Transfections, and Virus Recovery

DNA fragments were assembled using the Gibson Assembly Master Mix (New England BioLabs, Ipswich, MA, USA), as previously described [51]. Each assembly reaction contained three DNA fragments: the pUC19-F1-derived fragment, the pUC19-F2-derived fragment, and one of the pUC19-F3-derived fragments containing the donor xrRNA1 or xrRNA1-like element. Together, these fragments span the entire chimeric ZIKV genome and contain 30 bp overlaps that facilitate assembly. The assembled DNAs were transfected into C6/36 cells using Lipofectamine^TM^ 2000 Transfection Reagent (Thermo Fisher Scientific) and infectious chimeric viruses were recovered from transfected cells, as previously described [51].

### 2.6. RNA Extraction and Reverse Transcription-Polymerase Chain Reaction

Total RNA was extracted from cell monolayers and culture supernatants using Trizol Reagent (Thermo Fisher Scientific) and the QIAamp Viral RNA Mini Kit (Qiagen, Valencia, CA, USA), respectively. Complementary DNA was synthesized using SuperScript IV reverse transcriptase (Thermo Fisher Scientific) and PCR amplification was performed using Phusion high-fidelity DNA polymerase (Thermo Fisher Scientific). An aliquot of each PCR product was resolved on a 1% agarose gel and selected amplicons were purified using QIAquick Spin Columns (Qiagen). Purified amplicons were subjected to Sanger sequencing on a 3730xl DNA sequencer (Applied Biosystems, Foster City, CA, USA) at the Iowa State University DNA Facility.

### 2.7. Western Blot Analysis

Protein lysates were prepared from cell monolayers inoculated with wild-type or chimeric ZIKV and analyzed by Western blotting using an anti-ZIKV prM polyclonal antibody (GeneTex Inc., Irvine, CA, USA) or anti-β-actin polyclonal antibody (GeneTex Inc.), as previously described [51].

### 2.8. Growth Curves

Subconfluent monolayers of C6/36 and Vero cells in 175 cm^2^ flasks were inoculated with wild-type or chimeric ZIKV at a multiplicity of infection of 0.0001 and incubated for 1 h in cell culture media containing 2% fetal bovine sera, as previously described [50]. The inoculum was removed, the cells were washed twice with phosphate-buffered saline, and fresh media containing 2% fetal bovine sera was added. Aliquots of culture supernatant were collected daily for 7 days and titrated by plaque assay. Duplicate flasks were prepared for each virus, with each supernatant sample tested in triplicate. Data are presented as mean viral titers ± 1 standard deviation.

### 2.9. Plaque Morphology

Viral plaque morphology was assessed in Vero cells, as previously described [51]. Plaque diameters were measured using ImageJ software (version 1.54i; https://imagej.net/ij/), (accessed on 28 May 2024). Thirty plaques were measured for each virus.

### 2.10. Northern Blot Analysis

The production of sfRNAs in C6/36 and Vero cells inoculated with wild-type and chimeric ZIKV was assessed by Northern blot using standard protocols (Lifeasible, Shirley, NY, USA). Three DNA probes were generated. One probe targeted the ZIKV 3′ UTR downstream of the xrRNA1 element, whereas the remaining two targeted the 28S rRNA genes of *Ae. albopictus* and *C. sabaeus* (GenBank Accession Nos. KU501215.1, DQ397934.1, and XR_012092113.1, respectively). The DNA probes were generated by RT-PCR, and the primer sequences used for the probe synthesis are provided (Table 2). The relative abundance of individual sfRNA species was quantified by densitometric analysis of the Northern blot bands, and sfRNA1:sfRNA2 and sfRNA1:sfRNA3 ratios were calculated from the resulting band intensities. A single Northern blot analysis was performed for each virus, cell type, and probe; therefore, the resulting sfRNA ratios represent descriptive measurements and were not subjected to statistical analysis.

### 2.11. Structural Analysis of xrRNA1 Elements

The predicted secondary structures of the xrRNA1 and xrRNA1-like elements were generated using KnotFold and the resulting BPSEQ files were used to calculate structural metrics [62]. For each construct, structural predictions included the xrRNA1 or xrRNA1-like sequence together with 32 nt of flanking ZIKV sequence, comprising 16 nt immediately upstream and 16 nt immediately downstream of the substituted element. The sequence length (nt), total number of base pairs, pairing efficiency, number of crossing pairs, number of nested interactions, and number of structural stems were then determined. Pairing efficiency was calculated as (2 × total base pairs)/RNA length × 100. Crossing pairs were defined as base-pair interactions satisfying the relationship *i* < *k* < *j* < *l*, representing pseudoknot-type crossings. Nested interactions were defined as stems located within the loop region of another stem. Structural stems were defined as contiguous helical regions consisting of consecutive base pairs.

Topological approaches have previously been used to characterize RNA structural organization and complexity, particularly the arrangement of base-pairing interactions and pseudoknotted structures [63,64]. To provide an exploratory measure incorporating multiple features of the predicted xrRNA1 structures, a topological complexity score was developed for this study and calculated as (crossing pairs × 1.0) + (nested interactions × 2) + (structural stems × 0.5). The weighting factors were selected heuristically to integrate these structural features into a single comparative metric and were not derived from experimentally validated relationships between these features and xrRNA function. Accordingly, the topological complexity score was used as a descriptive comparative metric rather than as a validated predictor of xrRNA function. Minimum free energy (MFE) values were calculated using RNAfold (ViennaRNA package, v2.6.4), with more negative values indicating greater predicted thermodynamic stability. To integrate structural stability and topological complexity into a single metric, MFE values were multiplied by −1 so that larger values represented greater predicted stability. The transformed MFE values and topological complexity scores were standardized as z-scores across the 10 constructs and summed with equal weighting to generate a composite structural score, calculated as *z*(-MFE) + *z*(topological complexity). Higher composite structural scores indicate xrRNA1 or xrRNA1-like elements predicted to possess greater thermodynamic stability and greater structural complexity. The composite structural score was also developed specifically for this study as an exploratory descriptive metric and has not been independently validated as a predictor of xrRNA function.

### 2.12. Statistical Analysis

Statistical analyses were performed using GraphPad Prism 11.0.2. Viral growth curves are presented as mean viral titers ± 1 standard deviation. Plaque sizes of wild-type ZIKV and individual chimeric viruses were compared using unpaired two-tailed Welch’s *t*-tests, with adjustment for multiple comparisons. Plaque sizes were also compared among the nine chimeric viruses using Welch’s one-way ANOVA followed by Games-Howell post hoc tests for multiple pairwise comparisons. Adjusted *p* values < 0.05 were considered statistically significant.

## 3. Results

### 3.1. Sequence and Structural Analysis of xrRNA1 Elements

Flaviviral xrRNAs are classified into three structural groups, designated classes 1a, 1b, and 2, based on differences in their nucleotide sequences and RNA structures [36,40,41,42,44,45]. Class 1a xrRNAs are characteristic of MBFVs and dISFs, whereas class 1b xrRNAs occur in cISFs, pegiviruses, pestiviruses, and hepaciviruses, and class 2 xrRNAs are found in TBFVs and NKVs. Despite these sequence and structural differences, all three classes adopt compact tertiary conformations that stall XRN1 and promote sfRNA biogenesis.

The nucleotide sequences and predicted secondary structures of the xrRNA1 or xrRNA1-like elements from all viruses included in this study have been described previously [34,40,44], with the exception of LPKV. The putative LPKV xrRNA1 sequence was identified by aligning the LPKV 3′ UTR sequence with previously characterized xrRNA1 sequences from Binjari virus (a dISF) and selected MBFVs using the NCBI nucleotide alignment tool [32,35,42]. A candidate sequence that aligned closely with these xrRNA1 elements was identified at the corresponding position within the LPKV 3′ UTR. Alignment of this sequence with the xrRNA1 and xrRNA1-like elements of all viruses included in the study further supported its identification as the putative LPKV xrRNA1 element (Figure 1A). All xrRNA1 or xrRNA1-like elements contain five regions (α, β, γ, δ and ε) predicted to form stem structures, together with three nucleotides predicted to participate in base-triplet interactions (U-A-U in class 1a xrRNAs and C-G-C in class 1b and class 2 xrRNAs). The predicted secondary structure of LPKV xrRNA1 was manually modeled based on previously described class 1a xrRNA1 structures and closely resembles that of ZIKV xrRNA1 (Figure 1B).

### 3.2. Chimeric Virus Production

**Figure 2:**
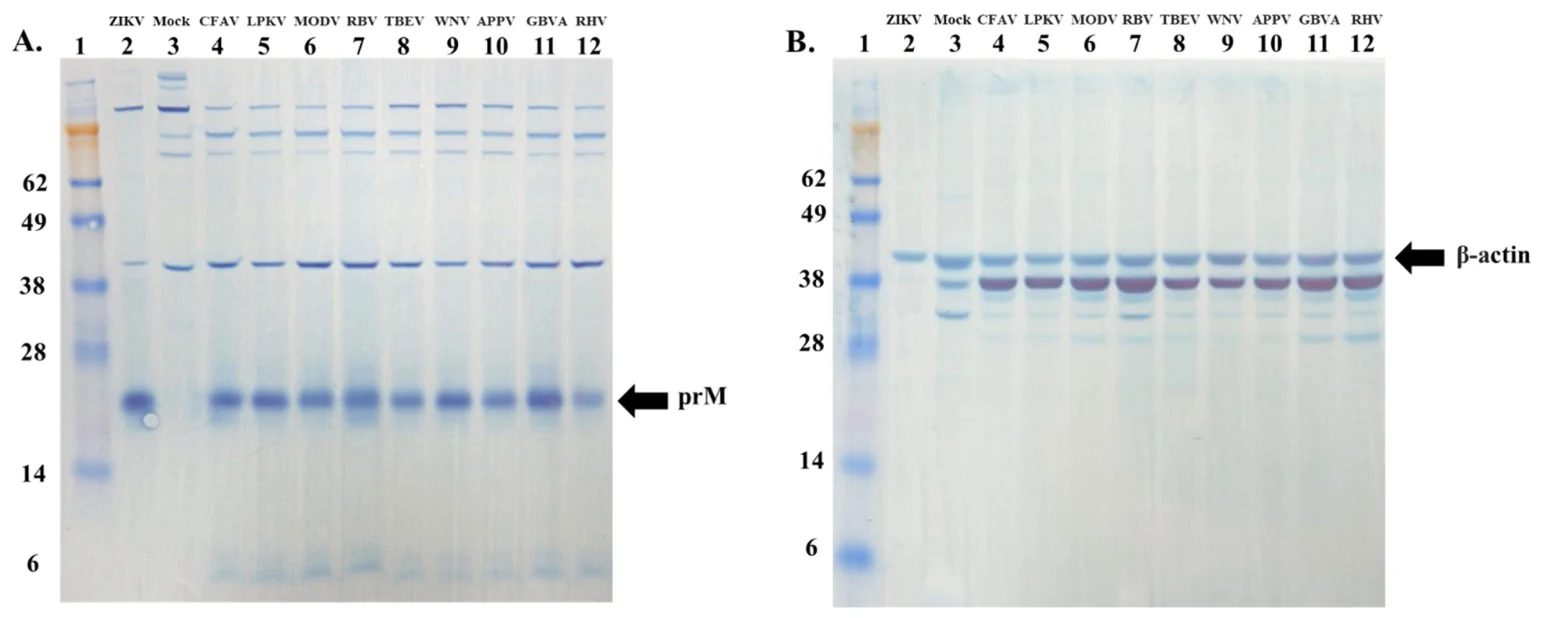

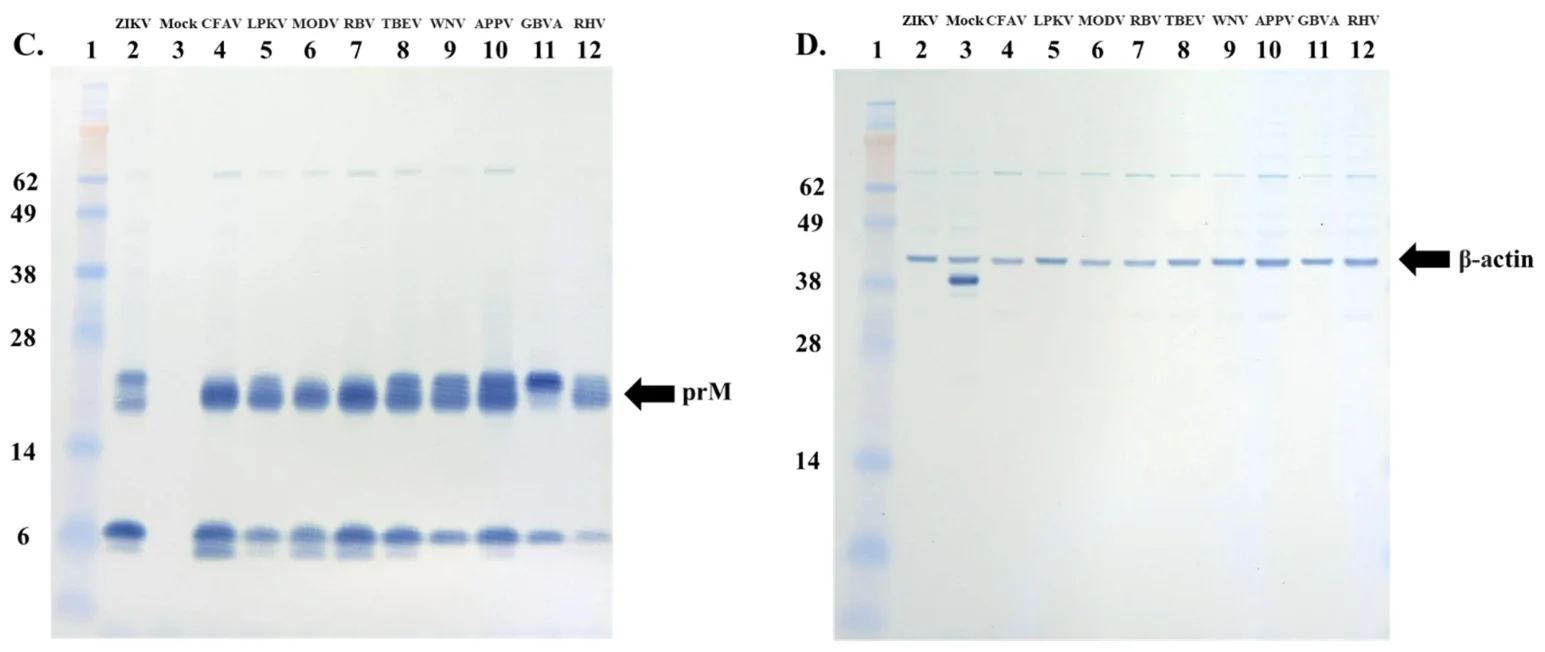
Western blot analysis demonstrates viral protein production following infection withall nine chimeric viruses in vertebrate and mosquito cells. Subconfluent monolayers of (**A**,**B**) Vero and (**C**,**D**) C6/36 cells were inoculated with wild-type ZIKV (lane 2) or chimeric viruses containing the xrRNA1 or xrRNA1-like elements of CFAV, LPKV, MODV, RBV, TBEV, WNV, APPV, GBVA or RHV (lanes 4 to 12, respectively) at a multiplicity of infection of 0.0001. Mock-inoculated cells (lane 3) were also included, as was a molecular weight marker (lane 1). Cell lysates were harvested at 5 days post-inoculation (Vero cells) or 7 days post-inoculation (C6/36 cells). Equal amounts of total protein were separated on 8–16% Tris-glycine gels and analyzed by Western blot using polyclonal antibodies against (**A**,**C**) ZIKV prM and (**B**,**D**) β-actin. Arrows indicate the expected migration positions of ZIKV prM (19 kDa) and β-actin (42 kDa). The abbreviation of the virus from which each xrRNA1 or xrRNA-1 element was derived is shown above the corresponding lane number.

Nine viral chimeras were constructed by replacing the ZIKV xrRNA1 element with the corresponding xrRNA1 or xrRNA1-like element from selected orthoflaviviruses (CFAV, LPKV, MODV, RBV, TBEV, and WNV), as well as from APPV (a pestivirus), GBVA (a pegivirus), and RHV (a hepacivirus). All chimeras produced infectious virus. Virus rescue was confirmed by passaging culture supernatants collected from the transfected cells at least twice in C6/36 or Vero cells and analyzing lysates from the final-passage cultures by Western blot using a ZIKV prM-specific antibody (Figure 2). Successful substitution of the xrRNA1 element was verified by RT-PCR and Sanger sequencing using primers annealing approximately 100 nt upstream and downstream of the substituted xrRNA1 element. No mutations were detected within any heterologous xrRNA1 or xrRNA1-like element. The resulting chimeric viruses were designated ZIKV-CFAV(xrRNA1), ZIKV-LPKV(xrRNA1), ZIKV-MODV(xrRNA1), ZIKV-RBV(xrRNA1), ZIKV-TBEV(xrRNA1), ZIKV-WNV(xrRNA1), ZIKV-APPV(xrRNA1), ZIKV-GBVA(xrRNA1), and ZIKV-RHV(xrRNA1), according to the virus from which the heterologous xrRNA1 or xrRNA1-like element was derived.

Western blot analysis revealed additional immunoreactive bands in some samples (Figure 2). In the prM blot of C6/36 cell lysates, an additional band of approximately 6 kDa was detected, consistent with the expected size of pr, which is generated by proteolytic cleavage of the prM precursor. Additional bands were also detected with the β-actin antibody, particularly in Vero cell lysates, and likely represent non-specific antibody reactivity.

### 3.3. In Vitro Replication Kinetics and Yields

**Figure 3:**
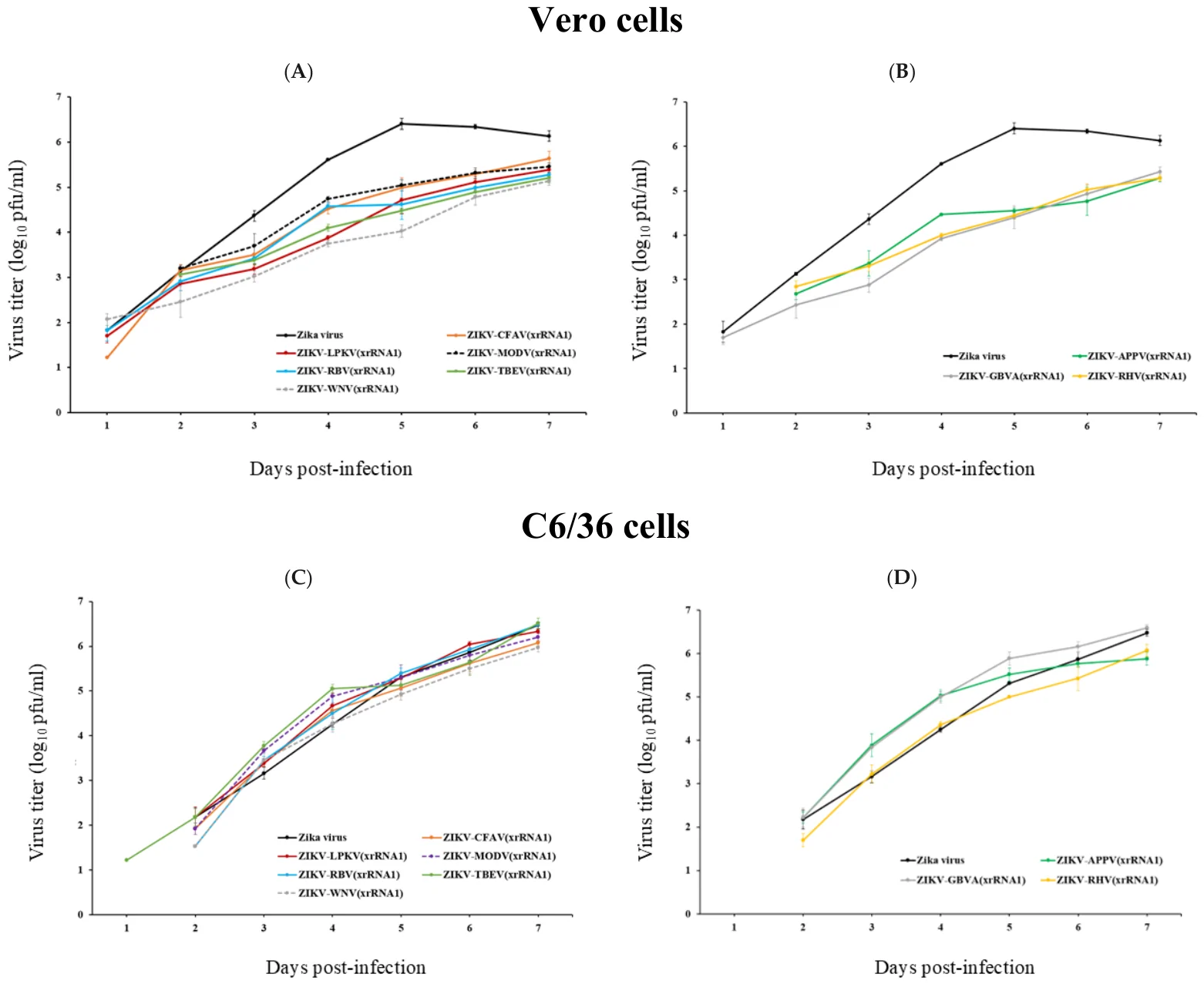
ZIKV replicated to higher titers than all chimeric viruses in Vero, but not C6/36, cells. Subconfluent monolayers of (**A**,**B**) Vero and (**C**,**D**) C6/36 cells grown in 175 cm^2^ flasks were inoculated with wild-type ZIKV or the chimeric viruses containing the xrRNA1 or xrRNA1-like elements of CFAV, LPKV, MODV, RBV, TBEV, WNV, APPV, GBVA or RHV at a multiplicity of infection of 0.0001. Supernatants were collected daily for 7 days and tested by plaque assay. The limit of detection of the plaque assay was 10 PFU/mL (1.0 log_10_ PFU/mL).

The replication kinetics and yields of wild-type ZIKV and the nine chimeric viruses were compared in Vero and C6/36 cells (Figure 3). In Vero cells, ZIKV consistently replicated to higher titers than all chimeric viruses. Differences became significant by day 2 post-infection (p.i.) for all but two chimeric viruses and by day 3 p.i. for all chimeric viruses, persisting through day 7 p.i. (Figure 3A,B, Table S1). In contrast, wild-type ZIKV showed no consistent replication advantage in C6/36 cells, where replication kinetics differed among viruses and over time (Figure 3B, Table S2). For example, on day 3 p.i., ZIKV titers were significantly lower than those of all chimeric viruses, except those containing RHV and WNV sequences. However, at later time points (days 5–7 p.i.), ZIKV replicated to significantly higher titers than several chimeric viruses, including those containing RHV and WNV sequences (Figure 3C,D; Table S2).

### 3.4. Plaque Morphologies

**Figure 4:**
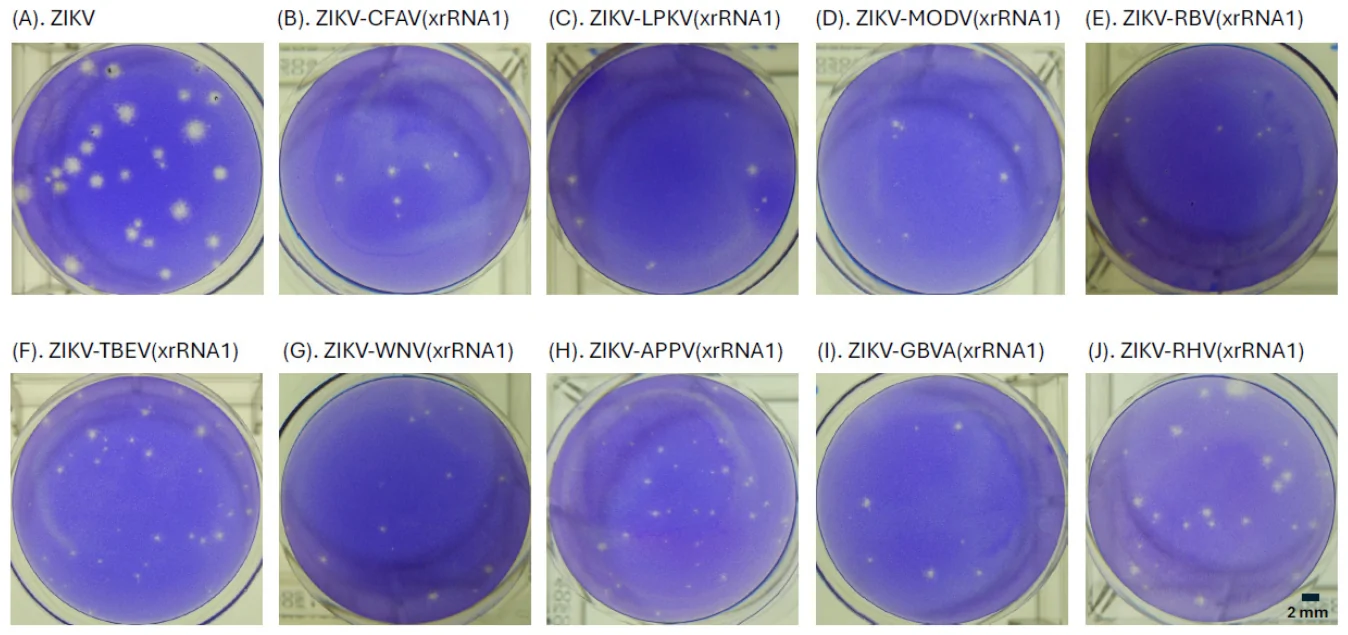
Plaque morphology comparisons reveal that ZIKV forms larger plaques than all nine chimeric viruses. Confluent monolayers of Vero cells in 35 mm^2^ culture dishes were inoculated with (**A**) wild-type ZIKV or the chimeric viruses containing the xrRNA1 or xrRNA1-like elements of (**B**) CFAV, (**C**) LPKV, (**D**) MODV, (**E**) RBV, (**F**) TBEV, (**G**) WNV, (**H**) APPV, (**I**) GBVA or (**J**) RHV at a multiplicity of infection of 0.0001. Following a 5-day incubation, cells were fixed and plaque diameters were measured, with a minimum of 30 plaques analyzed for each virus.

The plaque morphologies of the nine chimeric viruses were compared with those of wild-type ZIKV in Vero cells (Figure 4; Table 3). At 5 days p.i., ZIKV plaques had a mean diameter ± 1 standard deviation of 1.92 ± 0.64 mm, whereas the plaques produced by the chimeric viruses were approximately three- to five-fold smaller, ranging from 0.39 ± 0.23 mm for ZIKV-GBVA(xrRNA1) to 0.75 ± 0.58 mm for ZIKV-RHV(xrRNA1) (Table 3). All differences were statistically significant, as determined by unpaired two-tailed Welch’s *t*-test (all adjusted *p*-values ≤ 3 × 10^−10^). Plaque morphologies were also compared among the chimeric viruses (Table S3). Plaques produced by ZIKV-RHV(xrRNA1) were significantly larger than those produced by the chimeric viruses containing the xrRNA1 or xrRNA1-like elements of APPV, CFAV, GBVA, LPKV, RBV, and TBEV, as determined by Welch’s one-way ANOVA followed by Games-Howell post hoc testing (all adjusted *p* values ≤ 0.05). In addition, plaques produced by ZIKV-MODV(xrRNA1) were significantly larger than those of ZIKV-APPV(xrRNA1).

### 3.5. sfRNA Production

**Figure 5:**
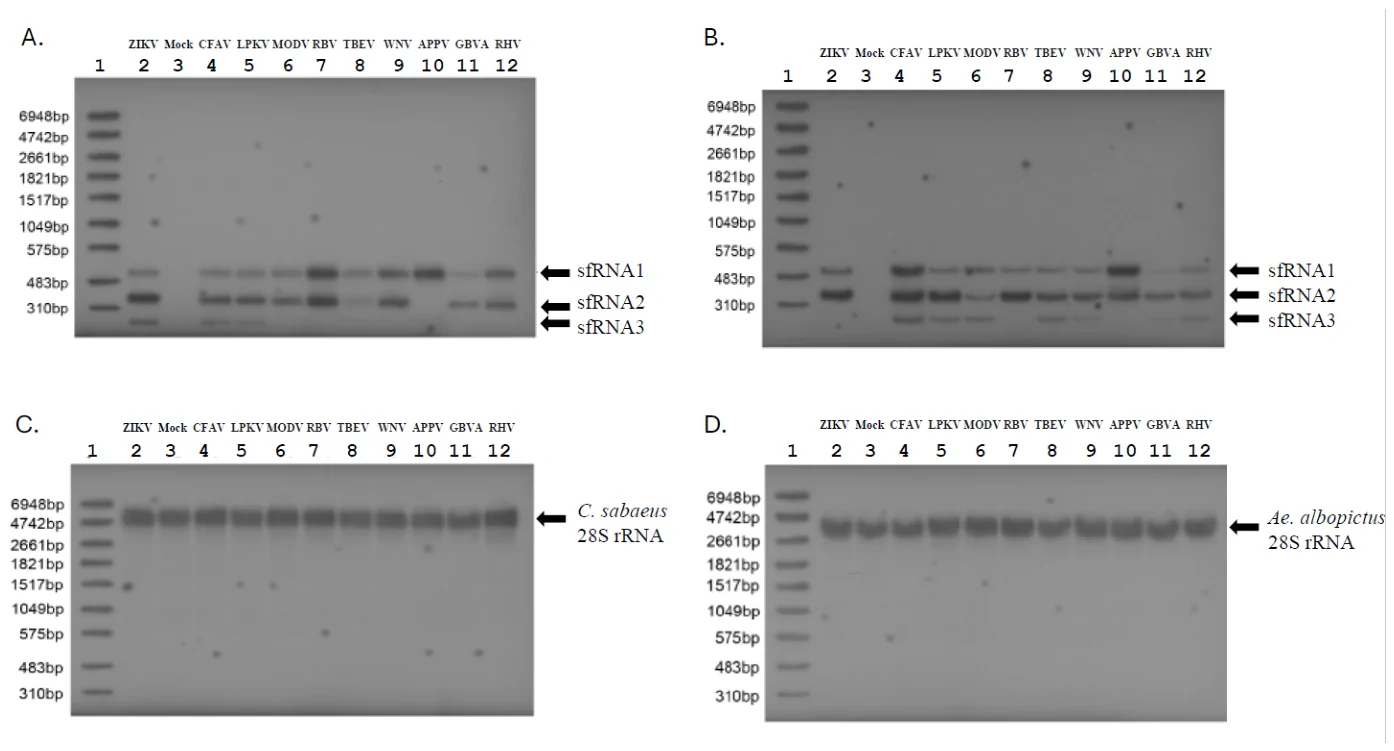
Northern blot analysis reveals that all nine chimeric viruses produce sfRNAs in Vero and C6/36 cells. Confluent monolayers of (**A**,**C**) Vero cells and (**B**,**D**) C6/36 cells grown in 175 cm^2^ flasks were inoculated with wild-type ZIKV (lane 2) or chimeric viruses containing the xrRNA1 or xrRNA1-like elements of CFAV, LPKV, MODV, RBV, TBEV, WNV, APPV, GBVA or RHV (lanes 4 to 12, respectively) at a multiplicity of infection of 0.0001 or they were mock-inoculated (lane 3). Cells were harvested at 4 days post-inoculation (Vero cells) or 6 days post-inoculation (C6/36 cells). Total RNA was extracted and analyzed by Northern blot using probes specific for (**A**,**B**) a region of the ZIKV genome located downstream of the xrRNA1 element, (**C**) *C. sabaeus* 28 rRNA or (**D**) *Ae. albopictus* 28 rRNA. Arrows indicate the positions of sfRNA1 (~500 nt.), sfRNA2 (~400 nt.), sfRNA3 (~260 nt.), *C. sabaeus* 28 rRNA (~4800 nt.), and *Ae. albopictus* 28 rRNA (4100 nt.). A molecular weight marker was included (lane 1). The abbreviation of the virus from which each xrRNA1 or xrRNA-1 element was derived is shown above the corresponding lane number.

Northern blot analysis was performed to compare the sfRNA profiles of wild-type ZIKV and the nine chimeric viruses in Vero and C6/36 cells (Figure 5). In Vero cells, wild-type ZIKV produced three sfRNA species with apparent sizes of approximately 500, 400, and 260 nt, corresponding to sfRNA1, sfRNA2, and sfRNA3, respectively (Figure 5A). Three sfRNA species were also detected in Vero cells infected with ZIKV-CFAV(xrRNA1) and ZIKV-LPKV(xrRNA1), the two chimeric viruses containing ISF-derived xrRNA1 elements. In contrast, only sfRNA1 and sfRNA2 were detected in Vero cells infected with the remaining chimeric viruses, except ZIKV-APPV(xrRNA1), in which only sfRNA1 was detected. In C6/36 cells, only sfRNA1 and sfRNA2 were detected in wild-type ZIKV-infected cells (Figure 5B). Likewise, only sfRNA1 and sfRNA2 were detected in cells infected with ZIKV-RBV(xrRNA1) and ZIKV-APPV(xrRNA1), whereas all three sfRNA species were detected in cells infected with the remaining seven chimeric viruses. Hybridization with 28S rRNA-specific probes confirmed that similar amounts of total RNA were loaded in each lane (Figure 5C,D).

To further compare sfRNA production among wild-type ZIKV and the chimeric viruses, the relative abundance of individual sfRNA species was quantified by calculating sfRNA1:sfRNA2 and sfRNA1:sfRNA3 ratios in Vero and C6/36 cells (Table 4). Wild-type ZIKV exhibited sfRNA1:sfRNA2 ratios of 0.37 and 0.50 in Vero and C6/36 cells, respectively. Most chimeric viruses also exhibited sfRNA1:sfRNA2 ratios of <1.0, indicating greater accumulation of sfRNA2 than sfRNA1. In contrast, ZIKV-RBV(xrRNA1), ZIKV-TBEV(xrRNA1), and ZIKV-RHV(xrRNA1) in Vero cells, and ZIKV-MODV(xrRNA1) and ZIKV-APPV(xrRNA1) in C6/36 cells, exhibited sfRNA1:sfRNA2 ratios ranging from 1.09 to 1.29, indicating that sfRNA1 and sfRNA2 accumulated at similar levels, with only a modest excess of sfRNA1.

Wild-type ZIKV exhibited an sfRNA1:sfRNA3 ratio of 0.54 in Vero cells (Table 4). Two chimeric viruses, ZIKV-CFAV(xrRNA1) and ZIKV-LPKV(xrRNA1), also produced detectable sfRNA3 in Vero cells, with sfRNA1:sfRNA3 ratios of 1.69 and 1.52, respectively. Wild-type ZIKV did not produce detectable sfRNA3 in C6/36 cells. In contrast, ZIKV-CFAV(xrRNA1) exhibited an sfRNA1:sfRNA3 ratio of 1.67 in C6/36 cells, indicating greater accumulation of sfRNA1 than sfRNA3. The remaining six chimeric viruses that produced detectable sfRNA3 exhibited sfRNA1:sfRNA3 ratios ranging from 0.79 to 1.27.

### 3.6. Predicted Structural Properties of the xrRNA1 Elements

Structural properties were calculated for each xrRNA1 or xrRNA1-like element, with 32 nt of flanking ZIKV sequence (16 nt upstream and 16 nt downstream of the substituted element) included in each analysis (Table 5). The parameters evaluated included sequence length, total number of base pairs, pairing efficiency, crossing pairs, nested interactions, structural stems, topological complexity, MFE, and a composite structural score incorporating MFE and topological complexity. Considerable variation was observed among the predicted structures. Sequence length ranged from 81 to 108 nt, topological complexity from 1.5 to 29.0, and MFE from −34.3 to −22.0 kcal/mol. Composite structural scores also varied substantially, ranging from −2.43 for CFAV to 2.93 for TBEV.

## 4. Discussion

The flaviviral 3′ UTR contains multiple highly structured RNA elements that regulate viral replication, translation, genome cyclization, and sfRNA production [21,65]. Previous studies have shown that exchanging the UTRs between MBFVs and dISFs can dramatically alter host range, demonstrating that untranslated genomic sequences are important determinants of flavivirus host specificity [51,60]. However, the contributions of individual RNA structures within these UTRs remain poorly understood. In the present study, we investigated whether xrRNA1 contributes to host specificity by replacing the ZIKV xrRNA1 element with xrRNA1 or xrRNA1-like elements from nine phylogenetically diverse members of the family *Flaviviridae*. All substitutions produced viable chimeric viruses that replicated in both vertebrate (Vero) and mosquito (C6/36) cells and retained the ability to produce sfRNA1.

Although all chimeric viruses replicated in both vertebrate and mosquito cells, replacement of the native ZIKV xrRNA1 element affected viral fitness. The consistent reduction in viral titers and plaque size in Vero cells suggests that xrRNA1 substitution influences virus–host cell interactions and associated cytopathic effects. The mechanisms underlying these phenotypes remain unknown, but the reduced titers and smaller plaques observed in Vero cells do not necessarily represent host restriction. Rather, they may reflect reduced compatibility of the heterologous xrRNA1 elements with the surrounding ZIKV genomic context, resulting in a general fitness cost that is particularly apparent in Vero cells. Such incompatibility could involve altered local or long-range RNA interactions, sfRNA production, or interactions with cellular factors required for efficient viral replication. The replication disadvantage observed in Vero cells was much less pronounced in C6/36 cells, where some chimeric viruses replicated less efficiently, whereas others replicated to titers similar to or exceeding those of wild-type ZIKV. This difference raises the possibility that the fitness consequences of heterologous xrRNA1 substitution are host cell-dependent. However, because only one vertebrate and one mosquito cell line were examined, our data do not establish whether the contrasting replication phenotypes reflect vertebrate- versus mosquito-specific requirements for xrRNA1 or differences particular to the cell lines examined. The possibility of host cell-dependent effects is consistent with a previous study demonstrating that a reporter ZIKV carrying mutations that abolished xrRNA1 replicated less efficiently than the wild-type reporter virus in vertebrate cells, but more efficiently in mosquito cells [47].

Because Vero cells are deficient in type I interferon production, our study does not address whether an intact interferon response influences the replication phenotypes associated with heterologous xrRNA1 substitution. Future studies using human cell lines with intact innate immune responses, such as A549 or Huh-7 cells, should help determine whether the effects of xrRNA1 substitution are cell type-dependent or influenced by antiviral responses. Interestingly, previous studies have reported differing results regarding the importance of xrRNA1 and sfRNA1 production during ZIKV replication in A549 cells. Pallarés et al. [47] reported that deletion of xrRNA1 reduced viral replication, whereas Sparks et al. [48] reported that disruption of xrRNA1-dependent sfRNA1 production did not significantly affect ZIKV replication in A549 cells. These differences may reflect the distinct genetic modifications employed and the resulting effects on sfRNA production. Another limitation of our study is that C6/36 cells lack a functional RNAi response [66]. Future studies using mosquito cell lines with an intact RNAi response, such as *Ae. aegypti* Aag2 cells, should help determine whether RNAi-mediated antiviral responses influence the replication phenotypes associated with xrRNA1 substitution.

As noted earlier, the replication phenotypes of the chimeric viruses in C6/36 cells were highly variable. Surprisingly, no apparent relationship to either the structural class of the heterologous xrRNA1 or xrRNA1-like element or the host range of the donor virus. For example, despite both containing class 1b xrRNA1-like elements, ZIKV-GBVA(xrRNA1) and ZIKV-RHV(xrRNA1) replicated to significantly higher and lower titers, respectively, than wild-type ZIKV at later time points. Likewise, the two chimeras containing ISF-derived xrRNA1 elements exhibited contrasting phenotypes, with ZIKV-LPKV(xrRNA1) reaching titers similar to those of wild-type ZIKV at later time points, whereas ZIKV-CFAV(xrRNA1) generally replicated less efficiently. These findings suggest that the phenotypic consequences of xrRNA1 substitution may depend on sequence-specific or context-dependent interactions between the substituted element and the surrounding ZIKV genome. However, qualitative comparison of the predicted structural properties with the observed replication phenotypes did not reveal an obvious pattern, suggesting that the structural parameters evaluated here do not readily account for the observed phenotypic differences. For example, MODV and TBEV had markedly different composite structural scores (0.43 and 2.93, respectively), yet the corresponding chimeric viruses exhibited very similar replication kinetics in C6/36 cells.

All heterologous xrRNA1 or xrRNA1-like elements supported sfRNA1 production in both mosquito and vertebrate cells. The donor elements represented all three structural classes and were derived from mosquito-borne, tick-borne, insect-specific, and vertebrate-specific orthoflaviviruses, as well as representative members of the *Hepacivirus*, *Pegivirus*, and *Pestivirus* genera. Although the ability of structurally diverse xrRNA elements to resist degradation by XRN1 and other 5′→3′ exoribonucleases has been demonstrated previously [41], our findings show that these evolutionarily divergent elements retain this function when introduced into the ZIKV genomic context, resulting in detectable sfRNA1 production in both vertebrate and mosquito cells.

Wild-type ZIKV exhibited distinct sfRNA profiles in vertebrate and mosquito cells, with three sfRNA species detected in Vero cells but only sfRNA1 and sfRNA2 detected in C6/36 cells. Similar cell type-dependent differences in sfRNA profiles have been reported previously. For example, all three sfRNA species were detected in Vero cells infected with ZIKV (strain Natal), whereas only sfRNA1 and sfRNA2 were observed in infected C6/36 cells [67]. Likewise, ZIKV (strain PRVABC59) produced three detectable sfRNA species in Vero and A549 cells, but only sfRNA1 and sfRNA2 in C6/36 cells [35]. Another study detected only sfRNA1 and sfRNA2 in A549 cells infected with the same ZIKV strain, although sfRNA3 was detectable after infection with an xrRNA1-deficient mutant virus [48]. Furthermore, only a single sfRNA species was detected in Vero cells infected with ZIKV (strain PE243) [68]. Similar variation in sfRNA profiles has been reported for other MBFVs [28,33]. Collectively, these studies, together with the present findings, demonstrate that sfRNA profiles are highly dynamic and can vary according to viral strain, host cell type, and experimental conditions.

The chimeric viruses also displayed diverse sfRNA profiles, with xrRNA1 substitution affecting the detection of downstream sfRNA2 and sfRNA3 in a sequence- and host cell-dependent manner. These findings suggest that the effects of xrRNA1 substitution are not necessarily confined to sfRNA1 production but can extend to downstream regions of the 3′ UTR. In Vero cells, all three sfRNA species were detected in cells infected with ZIKV-CFAV(xrRNA1) and ZIKV-LPKV(xrRNA1), consistent with wild-type ZIKV, whereas only sfRNA1 and sfRNA2 were detected in cells infected with the remaining chimeric viruses, except ZIKV-APPV(xrRNA1), in which only sfRNA1 was detected. The mechanisms underlying these differences are unclear, although the qualitative comparison revealed no apparent relationship between the observed phenotypes and xrRNA1 structural class or composite structural score. For example, despite both belonging to class 1b, the APPV and CFAV xrRNA1 elements were associated with markedly different sfRNA profiles, with the corresponding chimeric viruses producing only sfRNA1 and all three sfRNA species, respectively. Furthermore, the APPV and WNV xrRNA1 elements had similar composite structural scores (1.13 and 1.16, respectively), yet sfRNA2 was detected in Vero cells infected with ZIKV-WNV(xrRNA1), but not ZIKV-APPV(xrRNA1). It is interesting that sfRNA3 was observed only for the chimeric viruses containing ISF-derived xrRNA1 elements. One explanation is that the sequence or structural features unique to ISF-derived xrRNA1 elements favor the accumulation of sfRNA3. However, sfRNA profiles showed no consistent relationship with the host association of the donor virus. For example, sfRNA2 was not detected in Vero cells infected with ZIKV-APPV(xrRNA1), but was detected in Vero cells infected with all other chimeric viruses containing xrRNA1 or xrRNA1-like elements derived from vertebrate-infecting flaviviruses.

The sfRNA profiles observed in C6/36 cells differed markedly from those in Vero cells. Only sfRNA1 and sfRNA2 were detected in cells infected with ZIKV-RBV(xrRNA1) and ZIKV-APPV(xrRNA1), consistent with wild-type ZIKV, whereas all three sfRNA species were detected in cells infected with the remaining seven chimeric viruses. As in Vero cells, qualitative comparison revealed no apparent relationship between these phenotypes and xrRNA1 structural class, composite structural score, or host association of the donor virus. Instead, these findings suggest that sequence- or structure-dependent properties of individual xrRNA1 elements influence the accumulation of downstream sfRNA species in a host cell-dependent manner. Because xrRNA1 substitution altered the production of sfRNAs generated from downstream regions of the 3′ UTR, the effects of xrRNA1 replacement may extend beyond the substituted element itself. Heterologous xrRNA1 sequences could alter local or long-range RNA interactions within the 3′ UTR, thereby affecting the folding, accessibility, or XRN1 resistance of downstream RNA structures. The contrasting sfRNA profiles observed in Vero and C6/36 cells further suggest that these effects may also be influenced by interactions between viral RNA structures and host cellular factors.

The relative abundance of sfRNA species differed among the chimeric viruses. In Vero cells, most viruses produced more sfRNA2 than sfRNA1, although ZIKV-RBV(xrRNA1), ZIKV-TBEV(xrRNA1), and ZIKV-RHV(xrRNA1) produced slightly more of the larger sfRNA1 species. Likewise, in C6/36 cells, most chimeric viruses produced either more sfRNA1 than sfRNA2 or similar amounts of both sfRNA species. Multiple chimeric viruses failed to produce sfRNA3 in one or both cell types, but the sfRNA1:sfRNA3 ratios of those that produced it ranged from 0.79 to 1.69. Overall, these findings demonstrate that heterologous xrRNA1 substitution influences the relative abundance of sfRNA species, but qualitative comparison revealed no consistent relationship between sfRNA ratios and xrRNA1 structural class, composite structural score, or host association of the donor virus.

The inability to detect a particular sfRNA species does not exclude the possibility that it was produced at levels below the detection threshold of the Northern blot assay. In addition, sfRNA accumulation is dynamic during flavivirus infection, and the sfRNA profiles described here represent a single sampling time under the experimental conditions used. Digital droplet PCR analysis of A549 cells infected with ZIKV or each of the four dengue virus serotypes showed that sfRNA levels increased throughout infection and generally reached a plateau between 24 and 48 h post-infection (hpi) [38]. Northern blot analysis demonstrated that the relative abundance of individual sfRNA species also changed over time. For example, sfRNA2 was more abundant than sfRNA1 in dengue virus 2-infected A549 cells at 18 hpi, whereas the two species accumulated to similar levels at 36 and 48 hpi.

## 5. Conclusions

Our findings demonstrate that xrRNA1 and xrRNA1-like elements from highly divergent members of the *Flaviviridae* can function within the ZIKV genomic context, as their substitution for the native ZIKV xrRNA1 did not abolish virus production. Although replacement of the native ZIKV xrRNA1 element substantially reduced viral fitness in Vero cells, as shown by reduced replication efficiency and smaller plaque size, it did not abolish sfRNA1 production or change the *in vitro* host range relative to parental ZIKV. Previous studies have shown that replacement of the entire UTRs profoundly alters flavivirus host range. The finding that none of the xrRNA1 substitutions altered *in vitro* host range suggests that xrRNA1 is not an essential determinant of flavivirus host specificity under the experimental conditions used in this study. Because all experiments were performed in cultured cells, the effects of xrRNA1 substitution on viral fitness, sfRNA production, and host specificity in vivo remain unknown. Future studies examining other structural elements within the UTRs, individually or in combination, including simultaneous replacement of both xrRNA1 and xrRNA2, should provide further insight into the mechanisms by which the flaviviral UTRs regulate host adaptation and evolution.

## Figures and Tables

**Table 1:** Primers used for the construction of viral chimeras.

Primer Name ^a^	Primer Sequence (5′-3′)	Target	ZIKV Genomic Position
ZIKV-3F	CCCAGGAGAAGCTGGGAAACC	ZIKV 3′ UTR	10,450–10,470
ZIKV-3R	ACATTAAGATTGGTGCTTACA	ZIKV 3′ UTR	10,374–10,394
Chimera-1F	GGATCATGATGATAAACAATGTATGG	OpIE2-CA	N/A
Chimera-1R	CGGAACGACAGTGGGG	ZIKV NS1	3444–3459
Chimera-2F	GGAGTGCACAATGCCC	ZIKV NS1	3430–3445
Chimera-2R	AAGACGGACTATGTTCCACC	ZIKV NS5	8025–8044
Chimera-3F	CAAAGCTATGGGTGGAACATAG	ZIKV NS5	8015–8036
Chimera-3R	AACTTGTTTATTGCAGCTTATAATGG	SV40 polyadenylation signal	N/A

^a^ Primer names ending in F and R denote forward and reverse primers, respectively; 3′ UTR, 3′ untranslated region; N/A, not applicable; OpIE2-CA, *Orgyia pseudotsugata* multicapsid nucleopolyhedrosis virus immediate-early 2 promoter; SV40, simian virus 40; ZIKV, Zika virus.

**Table 2:** Primers used to generate the probes for the Northern blot analysis.

Primer Name ^a^	Primer Sequence (5′-3′)	Target	Expected Size of Amplicon (bp)
Zika virus-F	GAGAACGCCATGGCACGGAA	ZIKV 3′ UTR	263
Zika virus-R	CCAGCGTGGTGGAAACTCATG	ZIKV 3′ UTR	
Aedes 28S-F	CGCACCGATGGTAGAAGA	*Ae. albopictus* 28 rRNA	439
Aedes 28S-R	CTGCTAGAACACCACGTGCAGTA	*Ae. albopictus* 28 rRNA	
Vero 28S-F	CGAGCTCAGGGAGGACAGAA	*C. sabaeus* 28 rRNA	414
Vero 28S-R	CCTCAGCCAAGCACATACACCAA	*C. sabaeus* 28 rRNA	

^a^ Primer names ending in F and R denote forward and reverse primers, respectively, with each reverse primer used for both the reverse transcription and polymerase chain reaction; 3′ UTR, 3′ untranslated region; ZIKV, Zika virus.

**Table 3:** Mean diameters of plaques produced by Zika virus and the chimeric viruses containing heterologous xrRNA1 or xrRNA-like elements in Vero cells at 5 days post-infection ^a^.

Virus	Plaque Size in Millimeters (Mean Diameter ± 1 Standard Deviation)	Unpaired Two-Tailed Welch’s *t*-Test ^b^
ZIKV (wild-type)	1.92 ± 0.64	-
ZIKV-CFAV(xrRNA1)	0.59 ± 0.32	2.25 × 10^−13^
ZIKV-LPKV(xrRNA1)	0.60 ± 0.29	1.40 × 10^−13^
ZIKV-MODV(xrRNA1)	0.65 ± 0.42	1.1 × 10^−11^
ZIKV-RBV(xrRNA1)	0.57 ± 0.33	3.6 × 10^−14^
ZIKV-TBEV(xrRNA1)	0.41 ± 0.22	<1 × 10^−14^
ZIKV-WNV(xrRNA1)	0.48 ± 0.27	<1 × 10^−14^
ZIKV-APPV(xrRNA1)	0.39 ± 0.14	<1 × 10^−15^
ZIKV-GBVA(xrRNA1)	0.39 ± 0.23	<1 × 10^−14^
ZIKV-RHV(xrRNA1)	0.75 ± 0.58	3 × 10^−10^

^a^ Triplicate plaque assays were performed, with 10 plaques measured per virus/replicate; ^b^ Plaque sizes of Zika virus were compared to those of the chimeric viruses using an unpaired two-tailed Welch’s *t*-test.

**Table 4:** Relative abundance of sfRNA species in Vero and C6/36 cells infected with Zika virus and the chimeric viruses containing heterologous xrRNA1 or xrRNA1-like elements.

Virus	Relative Abundance of sfRNA Species ^a^			
	Vero		C6/36	
	sfRNA1:sfRNA2	sfRNA1:sfRNA3	sfRNA1:sfRNA2	sfRNA1:sfRNA3
CFAV	0.46	1.69	0.89	1.67
LPKV	0.48	1.52	0.38	0.96
MODV	0.57	- ^b^	1.17	1.27
RBV	1.09	-	0.35	-
TBEV	1.09	-	0.46	0.79
WNV	0.82	-	0.47	1.04
ZIKV	0.37	0.54	0.50	-
APPV	-	-	1.29	-
GBVA	0.50	-	0.26	0.79
RHV	1.14	-	0.55	0.92

^a^ A ratio > 1 indicates that sfRNA1 accumulated to higher levels than the smaller sfRNA species; ^b^ The smaller sfRNA species was not detected. Northern blots were performed once and therefore, the ratios should be considered semi-quantitative.

**Table 5:** Predicted structural properties of the xrRNA1 and xrRNA1-like elements.

Virus	Length ^a^ (nt)	Total Base Pairs	Pairing Efficiency (%)	Crossing Base Pairs	Nested Interactions	Structural Stems	Topological Complexity	MFE (kcal/mol)	Composite Structural Score ^b^
CFAV	81	12	29.6	0	0	3	1.5	−22.4	−2.43
LPKV	83	12	21.8	5	0	3	6.5	−25.9	−1.01
MODV	108	14	25.9	11	3	7	20.5	−25.9	0.43
RBV	106	17	32.1	18	2	9	26.5	−25	0.81
TBEV	107	14	26.2	23	1	8	29.0	−32.2	2.93
WNV	91	14	18.5	3	0	7	6.5	−34.3	1.16
ZIKV	86	16	37.2	7	5	8	21.0	−27.1	0.79
APPV	88	21	47.7	13	2	7	20.5	−28.6	1.13
GBVA	86	11	25.6	3	2	8	11.0	−22.0	−1.56
RHV	82	6	14.6	0	0	3	1.5	−23.1	−2.25

^a^ For each construct, structural predictions were performed by KnotFold using the xrRNA1 or xrRNA1-like sequence together with 32 nt of flanking or ZIKV sequence, comprising 16 nt immediately upstream and 16 nt immediately downstream of the substituted element; MFE: minimal free energy; ^b^ Composite structural scores are a computationally derived metric, not an experimentally validated measure of xrRNA1 function.

## Data Availability

All chimeric viruses generated in this study are available upon request. Raw data for the growth curve and plaque morphology experiments are available on FigShare (https://doi.org/10.6084/m9.figshare.33456088).

## Supplementary Materials

The following supporting information can be downloaded at: https://www.mdpi.com/article/10.3390/v18091023/s1, Table S1. *p*-values comparing ZIKV titers to chimeric virus titers over time in Vero cells; Table S2. *p*-values comparing ZIKV titers to chimeric virus titers over time in C6/36 cells; Table S3. *p* values for plaque size comparisons among chimeric viruses showing significant differences.
