# Supplementary material for: Chimeric Zika Viruses Containing xrRNA1 Elements of Divergent Flaviviruses Retain the Ability to Replicate in Vertebrate and Mosquito Cells and Produce Subgenomic Flaviviral RNA
Source: Viruses. 2026 Sep 15;18(9):1023. doi: 10.3390/v18091023 (PMC13611918; doi:10.3390/v18091023)
Supplement: Supplementary file 1 [file viruses-18-01023-s001.zip › viruses-4505808-supplementary.pdf]

SUPPLEMENTAL DATA

Table S1. P-values comparing ZIKV titers to chimeric virus titers over time in Vero cells

Chimeric virus	Number of days post-infection						
	1	2	3	4	5	6	7
	P-value[a]						
ZIKV-CFAV(xrRNA1)	1.7×10^{-3}	0.73	7.9×10^{-5}	6.7×10^{-7}	7.6×10^{-7}	3.8×10^{-8}	2.7×10^{-4}
ZIKV-LPKV(xrRNA1)	0.33	8.0×10^{-3}	7.5×10^{-9}	5.0×10^{-11}	7.5×10^{-6}	1.2×10^{-7}	1.9×10^{-6}
ZIKV-MODV(xrRNA1)	8.0×10^{-6}	4.2×10^{-2}	1.2×10^{-3}	3.7×10^{-8}	4.5×10^{-9}	4.0×10^{-7}	1.4×10^{-6}
ZIKV-RBV(xrRNA1)	1.00	5.0×10^{-2}	5.4×10^{-8}	5.6×10^{-8}	9.5×10^{-6}	1.4×10^{-9}	1.7×10^{-7}
ZIKV-TBEV(xrRNA1)	8.0×10^{-6}	0.28	4.2×10^{-8}	2.1×10^{-8}	5.8×10^{-9}	1.3×10^{-11}	8.2×10^{-8}
ZIKV-WNV(xrRNA1)	5.6×10^{-2}	4.3×10^{-3}	2.8×10^{-9}	1.9×10^{-9}	3.7×10^{-11}	8.3×10^{-7}	3.6×10^{-8}
ZIKV-APPV(xrRNA1)	8.0×10^{-6}	3.2×10^{-4}	1.1×10^{-4}	1.1×10^{-13}	2.2×10^{-8}	5.5×10^{-5}	2.2×10^{-7}
ZIKV-GBVA(xrRNA1)	0.33	1.8×10^{-3}	1.5×10^{-8}	6.1×10^{-11}	2.4×10^{-7}	5.2×10^{-6}	8.9×10^{-7}
ZIKV-RHV(xrRNA1)	8.0×10^{-6}	2.4×10^{-3}	3.2×10^{-8}	8.4×10^{-10}	2.8×10^{-10}	2.0×10^{-7}	4.0×10^{-7}

[a]Determined by Welch’s t-test, with $p \leq 0.05$ considered significant

Table S2. P-values comparing ZIKV titers to chimeric virus titers over time in C6/36 cells

Chimeric virus	Number of days post-infection						
	1	2	3	4	5	6	7
	P-value[a]						
ZIKV-CFAV(xrRNA1)	n/a[b]	3.2×10^{-2}	5.7×10^{-3}	2.8×10^{-5}	2.7×10^{-2}	3.7×10^{-2}	3.0×10^{-6}
ZIKV-LPKV(xrRNA1)	n/a	1.00	7.0×10^{-3}	1.3×10^{-2}	0.62	0.071	8.5×10^{-3}
ZIKV-MODV(xrRNA1)	n/a	3.2×10^{-2}	7.1×10^{-5}	4.4×10^{-5}	0.93	0.44	4.9×10^{-4}
ZIKV-RBV(xrRNA1)	n/a	5.9×10^{-4}	1.2×10^{-3}	0.16	0.13	0.46	0.79
ZIKV-TBEV(xrRNA1)	$<10^{-6}$	1.00	5.0×10^{-6}	1.2×10^{-7}	4.9×10^{-2}	0.14	0.51
ZIKV-WNV(xrRNA1)	n/a	5.9×10^{-4}	0.055	0.90	7.2×10^{-4}	2.5×10^{-3}	2.0×10^{-6}
ZIKV-APPV(xrRNA1)	n/a	0.70	5.5×10^{-4}	3.0×10^{-6}	1.8×10^{-2}	0.26	3.7×10^{-5}
ZIKV-GBVA(xrRNA1)	n/a	0.75	2.9×10^{-5}	3.9×10^{-6}	2.1×10^{-4}	9.3×10^{-3}	2.0×10^{-2}
ZIKV-RHV(xrRNA1)	n/a	1.4×10^{-3}	0.57	3.6×10^{-2}	2.4×10^{-7}	9.3×10^{-3}	5.0×10^{-4}

[a]Determined by Welch's t-test, with $p \leq 0.05$ considered significant; [b]not applicable (titer was below the limit of detection)

Table S3. P values for plaque size comparisons among chimeric viruses showing significant differences

Virus 1[a]	Virus 2[b]	Adjusted P value[c]
ZIKV-RHV(xrRNA1)	ZIKV-APPV(xrRNA1)	3.2×10^{-4}
ZIKV-RHV(xrRNA1)	ZIKV-CFAV(xrRNA1)	0.041
ZIKV-RHV(xrRNA1)	ZIKV-GBVA(xrRNA1)	4.5×10^{-3}
ZIKV-RHV(xrRNA1)	ZIKV-LPKV(xrRNA1)	0.038
ZIKV-RHV(xrRNA1)	ZIKV-RBV(xrRNA1)	0.044
ZIKV-RHV(xrRNA1)	ZIKV-TBEV(xrRNA1)	1.1×10^{-3}
ZIKV-MODV(xrRNA1)	ZIKV-APPV(xrRNA1)	0.031

[a]The chimeric virus producing the larger plaques; [b]The chimeric virus producing the smaller plaques; [c]Determined by Welch's one-way ANOVA followed by Games-Howell post hoc testing, with adjusted p values $\leq$ 0.05 considered significant.
